# Recombinant methioninase effectively targets a Ewing's sarcoma in a patient-derived orthotopic xenograft (PDOX) nude-mouse model

**DOI:** 10.18632/oncotarget.15823

**Published:** 2017-03-01

**Authors:** Takashi Murakami, Shukuan Li, Qinghong Han, Yuying Tan, Tasuku Kiyuna, Kentaro Igarashi, Kei Kawaguchi, Ho Kyoung Hwang, Kentaro Miyake, Arun S. Singh, Scott D. Nelson, Sarah M. Dry, Yunfeng Li, Yukihiko Hiroshima, Thinzar M. Lwin, Jonathan C. DeLong, Takashi Chishima, Kuniya Tanaka, Michael Bouvet, Itaru Endo, Fritz C. Eilber, Robert M. Hoffman

**Affiliations:** ^1^ AntiCancer, Inc., San Diego, California, USA; ^2^ Department of Surgery, University of California, San Diego, California, USA; ^3^ Department of Gastroenterological Surgery, Graduate School of Medicine, Yokohama City University, Yokohama, Japan; ^4^ Division of Hematology-Oncology, University of California, Los Angeles, California, USA; ^5^ Department of Pathology, University of California, Los Angeles, California, USA; ^6^ Division of Surgical Oncology, University of California, Los Angeles, California, USA

**Keywords:** recombinant methioninase, patient-derived orthotopic xenograft, Ewing's sarcoma, recalcitrant cancer, nude mice

## Abstract

Methionine dependence is due to the overuse of methionine for aberrant transmethylation reactions in cancer. Methionine dependence may be the only general metabolic defect in cancer. In order to exploit methionine dependence for therapy, our laboratory previously cloned L-methionine α-deamino-γ-mercaptomethane lyase [EC 4.4.1.11]). The cloned methioninase, termed recombinant methioninase, or rMETase, has been tested in mouse models of human cancer cell lines. Ewing's sarcoma is recalcitrant disease even though development of multimodal therapy has improved patients'outcome. Here we report efficacy of rMETase against Ewing's sarcoma in a patient-derived orthotopic xenograft (PDOX) model. The Ewing's sarcoma was implanted in the right chest wall of nude mice to establish a PDOX model. Eight Ewing's sarcoma PDOX mice were randomized into untreated control group (*n* = 4) and rMETase treatment group (*n* = 4). rMETase (100 units) was injected intraperitoneally (i.p.) every 24 hours for 14 consecutive days. All mice were sacrificed on day-15, 24 hours after the last rMETase administration. rMETase effectively reduced tumor growth compared to untreated control. The methionine level both of plasma and supernatants derived from sonicated tumors was lower in the rMETase group. Body weight did not significantly differ at any time points between the 2 groups. The present study is the first demonstrating rMETase efficacy in a PDOX model, suggesting potential clinical development, especially in recalcitrant cancers such as Ewing's sarcoma.

## INTRODUCTION

Methionine dependence is the enhanced requirement of methionine for cancer cells compared to normal cells. Methionine dependence may be the only known general and very widespread metabolic defect in cancer [1].

Out of 23 cell lines derived from diverse types of human tumors, 11 did not grow at all in methionine-depleted, homocysteine-containing (MET^−^HCY^+^) medium and were absolutely methionine-dependent, whereas 3 grew only slightly in this medium. Many of the cancer cell lines tested have little else in common other than the fact that they are methionine-dependent. The high frequency of occurrence of methionine dependence in diverse types of human cancer cells indicated that methionine dependence could be an important aspect of oncogenic transformation. Normal unestablished cell strains thus far characterized grow well in MET^−^HCY^+^ medium [2, 3].

Twenty-one different human tumor cell lines (4 lung, 4 colon, 4 kidney, 4 melanoma, 3 CNS, and 2 prostate) and normal cell strains were treated with recombinant methioninase (rMETase) *in vitro*. rMETase had a mean IC_50_ for the cancer cells, which was one order of magnitude lower than that for normal cell strains [4].

Many lines of evidence indicated that the malignant and transformed cells synthesize large amounts of methionine endogenously despite their requirement for elevated exogenous methionine [1].

In a diverse set of human cancer cell lines, all were found to be defective in at least one aspect of methionine metabolism, giving rise to the possibility of a general metabolic defect in cancer [5].

We observed that cancer cells have enhanced overall rates of transmethylation compared to normal human fibroblasts. The overuse of methionine for enhanced and unbalanced transmethylation may be the basis of the methionine dependence of cancer cells. The overuse of methionine by cancer cells is termed the “Hoffman effect”. The alteration of such a fundamental process as transmethylation in cancer may be indicative of its importance in the oncogenic process [6].

Rare cells from methionine-dependent cancer cell lines regained the normal ability to grow in MET^−^HCY^+^ medium [7]. Methionine-independent revertants had much lower basal transmethylation rates than parental methionine-dependent cell lines. These results further suggested that methionine dependence is due to an increase in the rate of transmethylation reactions [7].

By selecting for methionine independence, it is possible to select for cells, which became less malignant, indicating further a linkage between altered methionine metabolism and oncogenic transformation [7–9].

rMETase arrested growth of HCT 15 and HT29 colon cancer in nude mice for 1 week after treatment termination. Colo 205 and SW 620 colon cancer were partially arrested by rMETase [10].

Methionine dependence was also found to occur in fresh patient tumors histocultured on Gelfoam® [11].

Our laboratory pioneered the patient-derived orthotopic xenograft (PDOX) nude mouse model with the technique of surgical orthotopic implantation (SOI) [12, 13]. PDOX models were established from patients with colon [14–16], pancreatic [17–28], breast [29], ovarian [30], lung [31] and stomach cancer [32], and mesothelioma [33] in our laboratory, resulting in primary and metastatic tumor growth very similar to that of the patient [32]. Recently, PDOX models of sarcoma have been developed [34–38], as well as for cervical cancer [39–41] and melanoma [42–44] in our laboratory.

Ewing's sarcoma is a rare and aggressive malignancy. Previously, a tumor from a patient with a Ewing's sarcoma with cyclin-dependent kinase inhibitor 2A/B (CDKN2A/B) loss and FUS-ERG fusion was implanted in the right chest wall of nude mice to establish a PDOX model. The Ewing's sarcoma PDOX model was sensitive to the CDK4/6 inhibitor, palbociclib, and the IGF-R inhibitor, linsitinib. The PDOX tumor was resistant to doxorubicin as was the patient [37].

The present report demonstrates the efficacy of rMETase on the PDOX model of Ewing's sarcoma.

## RESULTS AND DISCUSSION

### Established Ewing's sarcoma PDOX tumor recapitulates the original patient tumor

**Figure 1:**
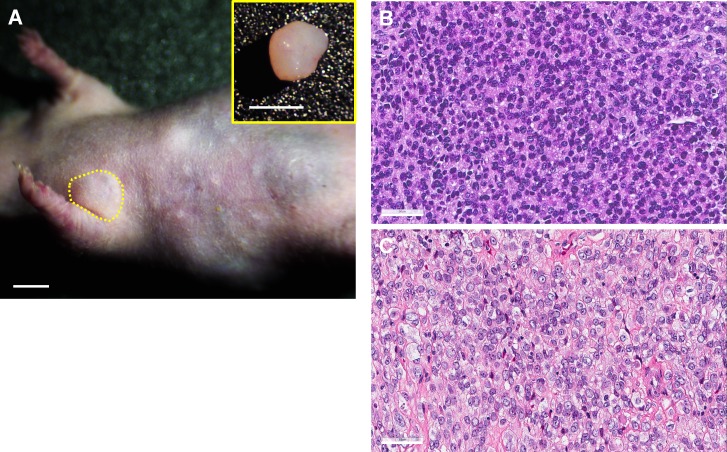
Histology of original and PDOX Ewing's sarcoma **A.** Four weeks after orthotopic tumor implantation, PDOX tumors grew in the right chest wall (yellow dotted line). Upper right panel shows the resected PDOX tumor. Scale bars: 5 mm. H&E staining of the PDOX tumor **B.** and original patient tumor **C.** Scale bars: 50 μm [37].

The PDOX tumor in the right chest wall of nude mice histologically recapitulated the original patient tumor (Figure 1) [37].

### Plasma L-methionine level was effectively decreased by rMETase intraperitoneal injection (i.p.) in nude mice

**Figure 2:**
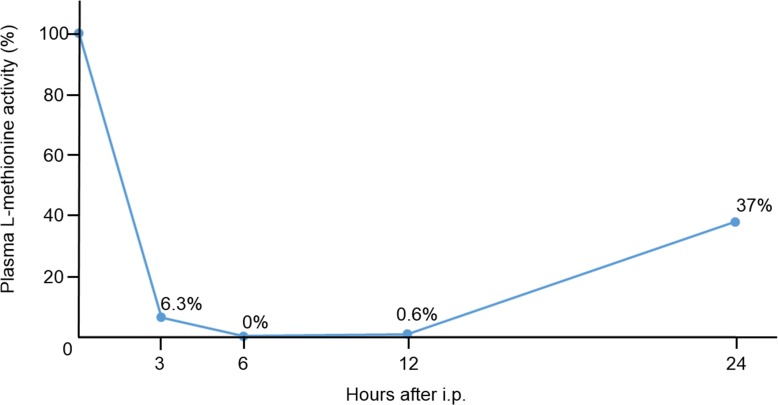
Pharmacodynamics study of plasma L-methionine level after intraperitoneal injection of rMETase Nude mice without tumors were used for the analysis. Plasma L-methionine levels were measured before injection, and 3, 6, 12, and 24 hours after i.p. injection of rMETase. The percentage of initial L-methionine level is indicated at each time point. L-methionine was measured by HPLC as described in the Materials and Methods. *N* = 1.

Plasma L-methionine levels decreased after 6 to 12 hours after i.p. administration of rMETase (Figure 2). Twenty-four hours after administration, plasma L-methionine level was still decreased to 37% of initial levels.

### Ewing's sarcoma PDOX tumor growth was suppressed by rMETase treatment

**Figure 3:**
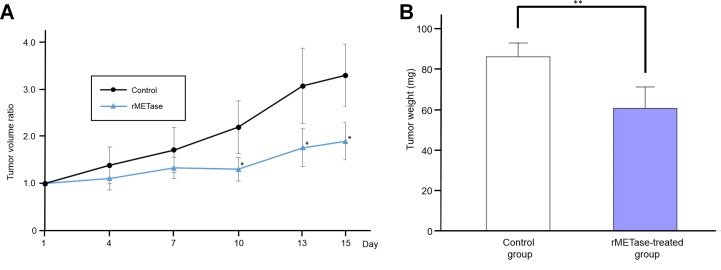
Response of Ewing's sarcoma PDOX to rMETase **A.** Line graph shows tumor volume ratio of both groups. Tumor volume ratio tumor volume at indicated tunes relative to tumor volume at day 0 in rMETase-treated group was significantly smaller from day10 to 15 compared to control. **B.** Bar graph shows resected tumor weight. Tumor weight was also significantly less in the rMETase-treated group than in the control group. **P < 0.01, *P < 0.05. Error bars: ± 1 SD. SD, standard deviation. *N* = 4 for each group.

**Figure 4:**
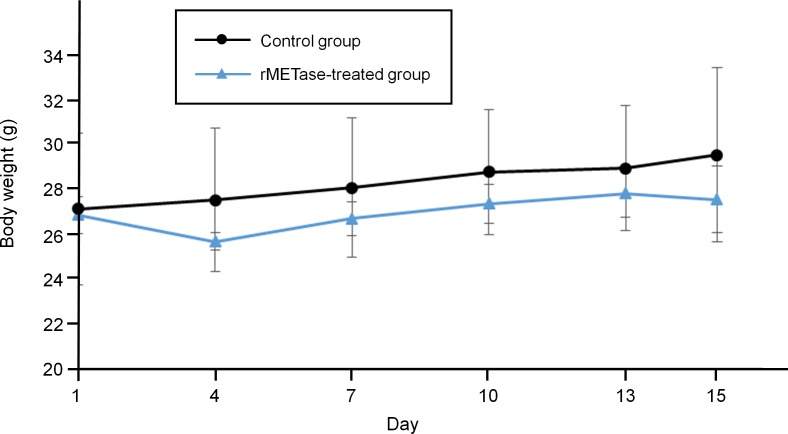
Response of Ewing's sarcoma PDOX mouse body weight to rMETase Line graphs show mouse body weight of each group. Error bars: ± 1 SD. *N* = 4 for each group.

The tumor volume ratio in the rMETase-treated group was significantly smaller from day 10 to 15 compared to the untreated control mice (*P* < 0.05) (Figure 3). Tumor weight was also significantly smaller in rMETase-treated group (60.6 ± 10.6 mg) than in control group (86.1 ± 6.8 mg) (*P* < 0.01) (Figure 3). Mouse body weight was not significantly reduced in the rMETase group (Figure 4).

### Plasma and tumor-extract L-methionine was reduced by rMETase

**Figure 5:**
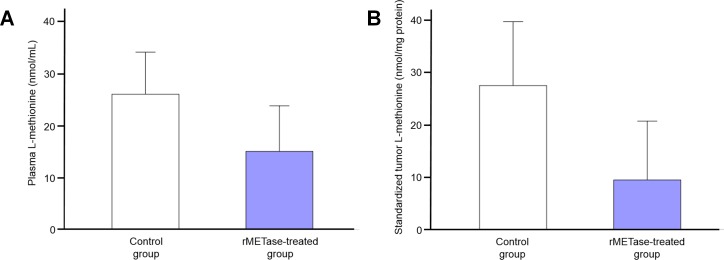
Plasma and Ewing's sarcoma PDOX tumor L-methionine levels at termination of the experiments Bar graphs show **A.** plasma L-methionine level and **B.** Tumor L-methionine level standardized by tumor protein concentration. Both plasma and tumor L-methionine levels in the rMETase-treated animals were lower than control. Error bars: ± 1 SD. *N* = 4 for each group.

Plasma L-methionine level in the rMETase-treated Ewing's sarcoma PDOX (15.0 ± 8.8 nmol/ml) trended to be lower than in the untreated control (26.0 ± 8.1 nmol/ml). L-methionine levels were reduced in the rMETase-treated tumors (9.5 ± 11.3 nmol/mg protein) compared to the untreated control tumor (27.5 ± 12.2 nmol/mg protein) (Figure 5). Although changes in methionine levels did not reach statistical significance, they show a definite trend of depletion. It should be noted that the methionine levels were measured 24 hours after the last rMETase administration during which time MET levels could have increased from both endogenous synthesis and mouse chow.

The present study has important implications since it is the first *in vivo* efficacy study of rMETase on a patient tumor, in this case, a PDOX model of Ewing's sarcoma, a recalcitrant cancer. The present results and previous results indicating the generality of methionine dependence, and the linkage of methionine dependence to fundamental properties of malignancy, suggest that rMETase is a specific tumor-targeting agent that may be applicable to the treatment of multiple types of cancer [1–10, 45–50]. Further rMETase efficacy studies in PDOX models of various tumor types should serve as a bridge to the clinic.

Previously-developed concepts and strategies of highly selective tumor targeting can take advantage of molecular targeting of tumors, including tissue-selective therapy which focuses on unique differences between normal and tumor tissues [51–57].

## CONCLUSIONS

The present study has demonstrated efficacy of rMETase on a Ewing's sarcoma PDOX model. This is the first experiment in which rMETase was tested on a patient-derived tumor in a mouse model. The results indicate the potential of rMETase in the clinic. However, important questions remain including whether rMETase can shrink tumors as monotherapy or whether combination with chemotherapy would be more effective. Combination of rMETase and cell-cycle-specific drugs could take advantage of the late-S/G_2_ cell arrest observed in cancer cells after methionine depletion [45–48, 58]. Another question remains what is the long-term fate of cancer cells arrested in late-S/G_2_ by rMETase such as whether the cells eventually undergo apoptosis or necrosis.

## MATERIALS AND METHODS

### Mice

Athymic male *nu/nu* nude mice (AntiCancer Inc., San Diego, CA), 4-6 weeks old, were used in this study. All animal studies were conducted in accordance with the principals and procedures outlined in the Guide for the Care and Use of Laboratory Animals, 8^th^ edition under Public Health Service Assurance Number A3873-1. Animals were housed with no more than 5 per cage and housed in a barrier facility on a high efficiency particulate arrestance (HEPA)-filtered rack under standard conditions of 12-hour light/dark cycles. The animals were fed an autoclaved laboratory rodent diet.

### Patient-derived Ewing's sarcoma tumor and establishment of PDOX

A female patient was diagnosed with Ewing's sarcoma in the right chest wall. She underwent surgery in the Department of Surgery, University of California, Los Angeles (UCLA). Written informed consent was obtained from the patient, and the Institutional Review Board (IRB) of UCLA approved this experiment. After mice were anethesized, a 7-mm skin incision was made on the right chest wall. A single tumor fragment (4 mm^3^) was then implanted orthotopically into the layer between pectoral muscle and intercostal muscle in right chest wall of nude mouse to establish the PDOX model [37].

### rMETase production

The pAC-1 rMETase high-expression clone was used for rMETase production. The fermentation procedure for host *E.coli* cells and the purification protocol for rMETase were the same as previously described: rMETase was purified by 3 different steps using columns of DEAE Sepharose FF and Sephacryl S-200HR, and ActiClean Etox, which is designed for eliminating endotoxin [49].

### Treatment dose and schedule

The PDOX model mice (*n* = 8) were randomized into an rMETase treatment group (*n* = 4) and an untreated control group (*n* = 4) when tumor volume reached 60 mm^3^. rMETase (100 units) was administered by intraperitoneal injection, every 24 hours for 14 consecutive days. Blood samples were collected at 24 hours after the last rMETase administration at which point the mice were sacrificed. Tumor length, width, and mouse body weight were measured twice a week. Tumor volume was calculated by the following formula: Tumor volume (mm^3^) = length (mm) × width (mm) × width (mm) × 1/2. Tumor volume ratio was defined as the ratio of volume on each measurement day relative to day 0.

### Plasma L-methionine levels

A mouse without tumor was treated with 100 units rMETase by i.p. administration. Fifty μl blood was collected from the retro-orbital plexus with heparinated capillary tubes before treatment and 3, 6, 12, and 24 hours after treatment. Plasma was obtained from the blood by centrifugation at 4,000 rpm for 5 minutes. Plasma L-methionine levels were measured with an HPLC (Hitachi L-6200A Intelligent pump; Hitachi, Ltd., Tokyo, Japan) after derivatization of serum amino acids with the fluoraldehyde reagent OPA as described previously [49, 50]. L-methionine levels are shown as the percentage of the initial plasma L-methionine level.

### Tumor protein level measurement

To standardize tumor L-methionine measurements, tumor protein levels were measured. Briefly, each PDOX tumor was placed in phosphate buffered saline (PBS) (1 ml). Tumors were sonicated on ice for 30 seconds and subsequently centrifuged at 12,000 rpm for 10 minutes. Supernatants were diluted to concentrations ranging from 200 to 1500 μl/ml. Protein assay reagent (Bio-Rad, Hercules, CA) was prepared as a 4-fold dilution and added to each tube with the sonicated tumor supernatents, then absorbance of 595 nm was measured with a U-2000 spectorophotometer (Hitachi, Tokyo, Japan). Protein levels were calculated from the standard curve obtained by protein standard, bovine serum albumin (BSA).

### Tumor L-methionine level analysis at termination

Tumor supernatants obtained from the above-described sonication procedure were precipitated by acetonitrile. Using the same procedure described above, L-methionine levels were determined as nmol/ml with the HPLC procedure described above [50]. Standardized L-methionine levels were calculated using the following formula: standardized L-methionine level (nmol/mg protein) = L-methionine level (nmol/ml) / protein level (mg protein/ml).

### Histological analysis

Fresh tumor samples were fixed in 10% formalin and embedded in paraffin before sectioning and staining. Tissue sections (5 μm) were deparaffinized in xylene and rehydrated in an ethanol series. Hematoxylin and eosin (H&E) staining was performed according to a standard protocol. Histological analysis was performed with a BHS system microscope (Olympus Corp., Tokyo, Japan). Images were acquired with INFINITY ANALYZE software (Lumenera Corporation, Ottawa, Canada) [37].

### Statistical analysis

SPSS statistics version 21.0 was used for all statistical analyses (IBM, New York City, NY, USA). Significant differences for continuous variables were determined using the Student's t-test. Both line graphs and bar graphs express mean values. An error bar shows standard deviation (SD). A probability value (*P*) was calculated between the control and treatment groups. *P* < 0.05 was considered statistically significant.

## References

[1] Hoffman RM (2015). Development of recombinant methioninase to target the general cancer-specific metabolic defect of methionine dependence: a 40-year odyssey. Expert Opin Biol Ther.

[2] Hoffman RM (1984). Altered methionine metabolism, DNA methylation and oncogene expression in carcinogenesis. A review and synthesis. Biochim Biophys Acta.

[3] Mecham JO, Rowitch D, Wallace CD, Stern PH, Hoffman RM (1983). The metabolic defect of methionine dependence occurs frequently in human tumor cell lines. Biochem Biophys Res Commun.

[4] Tan Y, Xu M, Hoffman RM (2010). Broad selective efficacy of recombinant methioninase and polyethylene glycol-modified recombinant methioninase on cancer cells *in vitro*. Anticancer Res.

[5] Stern PH, Wallace CD, Hoffman RM (1984). Altered methionine metabolism occurs in all members of a set of diverse human tumor cell lines. J Cell Physiol.

[6] Stern PH, Hoffman RM (1984). Elevated overall rates of transmethylation in cell lines from diverse human tumors. In Vitro.

[7] Judde JG, Ellis M, Frost P (1989). Biochemical analysis of the role of transmethylation in the methionine dependence of tumor cells. Cancer Res.

[8] Hoffman RM, Jacobsen SJ, Erbe RW (1979). Reversion to methionine independence in simian virus 40-transformed human and malignant rat fibroblasts is associated with altered ploidy and altered properties of transformation. Proc Natl Acad Sci USA.

[9] Hoffman RM (2015). Development of recombinant methioninase to target the general cancer-specific metabolic defect of methionine dependence: a 40-year odyssey. Expert Opin Biol Ther.

[10] Tan Y, Sun X, Xu M, Tan XZ, Sasson A, Rashidi B, Han Q, Tan XY, Wang X, An Z, Sun FX, Hoffman RM (1999). Efficacy of recombinant methioninase in combination with cisplatin on human colon tumors in nude mice. Clin Cancer Res.

[11] Freeman AE, Hoffman RM (1986). *In vivo*-like growth of human tumors *in vitro*. Proc Natl Acad Sci USA.

[12] Hoffman RM (2015). Patient-derived orthotopic xenografts: better mimic of metastasis than subcutaneous xenografts. Nature Rev Cancer.

[13] Hoffman RM (1999). Orthotopic metastatic mouse models for anticancer drug discovery and evaluation: a bridge to the clinic. Investigational New Drugs.

[14] Fu X, Besterman JM, Monosov A, Hoffman RM (1991). Models of human metastatic colon cancer in nude mice orthotopically constructed by using histologically intact patient specimens. Proc Natl Acad Sci USA.

[15] Metildi CA, Kaushal S, Luiken GA, Talamini MA, Hoffman RM, Bouvet M (2014). Fluorescently-labeled chimeric anti-CEA antibody improves detection and resection of human colon cancer in a patient-derived orthotopic xenograft (PDOX) nude mouse model. J Surg Oncol.

[16] Hiroshima Y, Maawy A, Metildi CA, Zhang Y, Uehara F, Miwa S, Yano S, Sato S, Murakami T, Momiyama M, Chishima T, Tanaka K, Bouvet M (2014). Successful fluorescence-guided surgery on human colon cancer patient-derived orthotopic xenograft mouse models using a fluorophore-conjugated anti-CEA antibody and a portable imaging system. J Laparoendosc Adv Surg Tech A.

[17] Fu X, Guadagni F, Hoffman RM (1992). A metastatic nude-mouse model of human pancreatic cancer constructed orthotopically from histologically intact patient specimens. Proc Natl Acad Sci USA.

[18] Kaushal S, McElroy MK, Luiken GA, Talamini MA, Moossa AR, Hoffman RM, Bouvet M (2008). Fluorophore-conjugated anti-CEA antibody for the intraoperative imaging of pancreatic and colorectal cancer. J Gastrointest Surg.

[19] Suetsugu A, Katz M, Fleming J, Moriwaki H, Bouvet M, Saji S, Hoffman RM (2012). Multi-color palette of fluorescent proteins for imaging the tumor microenvironment of orthotopic tumorgraft mouse models of clinical pancreatic cancer specimens. J Cell Biochem.

[20] Suetsugu A, Katz M, Fleming J, Truty M, Thomas R, Saji S, Moriwaki H, Bouvet M, Hoffman RM (2012). Imageable fluorescent metastasis resulting in transgenic GFP mice orthotopically implanted with human-patient primary pancreatic cancer specimens. Anticancer Res.

[21] Suetsugu A, Katz M, Fleming J, Truty M, Thomas R, Saji S, Moriwaki H, Bouvet M, Hoffman RM (2012). Non-invasive fluorescent-protein imaging of orthotopic pancreatic-cancer-patient tumorgraft progression in nude mice. Anticancer Res.

[22] Hiroshima Y, Maawy A, Sato S, Murakami T, Uehara F, Miwa S, Yano S, Momiyama M, Chishima T, Tanaka K, Bouvet M, Endo I, Hoffman RM (2014). Hand-held high-resolution fluorescence imaging system for fluorescence-guided surgery of patient and cell-line pancreatic tumors growing orthotopically in nude mice. J Surg Res.

[23] Hiroshima Y, Zhao M, Maawy A, Zhang Y, Katz MH, Fleming JB, Uehara F, Miwa S, Yano S, Momiyama M, Suetsugu A, Chishima T, Tanaka K (2014). Efficacy of Salmonella typhimurium A1-R versus chemotherapy on a pancreatic cancer patient-derived orthotopic xenograft (PDOX). J Cell Biochem.

[24] Hiroshima Y, Maawy A, Zhang Y, Murakami T, Momiyama M, Mori R, Matsuyama R, Katz MH, Fleming JB, Chishima T, Tanaka K, Ichikawa Y, Endo I (2014). Metastatic recurrence in a pancreatic cancer patient derived orthotopic xenograft (PDOX) nude mouse model is inhibited by neoadjuvant chemotherapy in combination with fluorescence-guided surgery with an anti-CA 19-9-conjugated fluorophore. Plos One.

[25] Hiroshima Y, Zhang Y, Murakami T, Maawy AA, Miwa S, Yamamoto M, Yano S, Sato S, Momiyama M, Mori R, Matsuyama R, Chishima T, Tanaka K (2014). Efficacy of tumor-targeting Salmonella typhimurium A1-R in combination with anti-angiogenesis therapy on a pancreatic cancer patient-derived orthotopic xenograph (PDOX) and cell line mouse models. Oncotarget.

[26] Hiroshima Y, Maawy AA, Katz MH, Fleming JB, Bouvet M, Endo I, Hoffman RM (2015). Selective efficacy of zoledronic acid on metastasis in a patient-derived orthotopic xenograph (PDOX) nude-mouse model of human pancreatic cancer. J Surg Oncol.

[27] Hiroshima Y, Maawy A, Zhang Y, Murakami T, Momiyama M, Mori R, Matsuyama R, Chishima T, Tanaka K, Ichikawa Y, Endo I, Hoffman RM, Bouvet M (2015). Fluorescence-guided surgery, but not bright-light surgery, prevents local recurrence in a pancreatic cancer patient-derived orthotopic xenograft (PDOX) model resistant to neoadjuvant chemotherapy (NAC). Pancreatology.

[28] Yano S, Hiroshima Y, Maawy A, Kishimoto H, Suetsugu A, Miwa S, Toneri M, Yamamoto M, Katz MHG, Fleming JB, Urata Y, Tazawa H, Kagawa S (2015). Color-coding cancer and stromal cells with genetic reporters in a patient-derived orthotopic xenograft (PDOX) model of pancreatic cancer enhances fluorescence-guided surgery. Cancer Gene Therapy.

[29] Fu X, Le P, Hoffman RM (1993). A metastatic-orthotopic transplant nude mouse model of human patient breast cancer. Anticancer Res.

[30] Fu X, Hoffman RM (1993). Human ovarian carcinoma metastatic models constructed in nude mice by orthotopic transplantation of histologically-intact patient specimens. Anticancer Res.

[31] Wang X, Fu X, Hoffman RM (1992). A new patient-like metastatic model of human lung cancer constructed orthotopically with intact tissue via thoracotomy in immunodeficient mice. Int J Cancer.

[32] Furukawa T, Kubota T, Watanabe M, Kitajima M, Fu X, Hoffman RM (1993). Orthotopic transplantation of histologically intact clinical specimens of stomach cancer to nude mice: correlation of metastatic sites in mouse and individual patient donors. Int J Cancer.

[33] Astoul P, Wang X, Colt HG, Boutin C, Hoffman RM (1996). A patient-like human malignant pleural mesothelioma nude-mouse model. Oncology Reports.

[34] Hiroshima Y, Zhao M, Zhang Y, Zhang N, Maawy A, Murakami T, Mii S, Uehara F, Yamamoto M, Miwa S, Yano S, Momiyama M, Mori R (2015). Tumor-targeting Salmonella typhimurium A1-R arrests a chemo-resistant patient soft-tissue sarcoma in nude mice. Plos One.

[35] Murakami T, DeLong J, Eilber FC, Zhao M, Zhang Y, Zhang N, Singh A, Russell T, Deng S, Reynoso J, Quan C, Hiroshima Y, Matsuyama R (2016). Tumor-targeting Salmonella typhimurium A1-R in combination with doxorubicin eradicate soft tissue sarcoma in a patient-derived orthotopic xenograft PDOX model. Oncotarget.

[36] Kiyuna T, Murakami T, Tome Y, Kawaguchi K, Igarashi K, Zhang Y, Zhao M, Li Y, Bouvet M, Kanaya F, Singh A, Dry S, Eilber FC (2016). High efficacy of tumor-targeting Salmonella typhimurium A1-R on a doxorubicin- and dactolisib-resistant follicular dendritic-cell sarcoma in a patient-derived orthotopic xenograft PDOX nude mouse model. Oncotarget.

[37] Murakami T, Singh AS, Kiyuna T, Dry SM, Li Y, James AW, Igarashi K, Kawaguchi K, DeLong JC, Zhang Y, Hiroshima Y, Russell T, Eckardt MA (2016). Effective molecular targeting of CDK4/6 and IGF-1R in a rare FUS-ERG fusion CDKN2A-deletion doxorubicin-resistant Ewing's sarcoma in a patient-derived orthotopic xenograft (PDOX) nude-mouse model. Oncotarget.

[38] Kiyuna T, Murakami T, Tome Y, Igarashi K, Kawaguchi K, Russell T, Eckhardt MA, Crompton J, Singh A, Bernthal N, Bukata S, Federman N, Kanaya F (2017). Labeling the stroma of a patient-derived orthotopic xenograft (PDOX) mouse models of undifferentiated pleomorphic soft-tissue sarcoma with red fluorescent protein for rapid non-invasive drug screening. J Cell Biochem.

[39] Hiroshima Y, Zhang Y, Zhang M, Maawy A, Mii S, Yamamoto M, Uehara F, Miwa S, Yano S, Murakami T, Momiyama M, Chishima T, Tanaka K (2015). Establishment of a patient-derived orthotopic xenograph (PDOX) model of HER-2-positive cervical cancer expressing the clinical metastatic pattern. Plos One.

[40] Hiroshima Y, Maawy A, Zhang Y, Zhang N, Murakami T, Chishima T, Tanaka K, Ichikawa Y, Bouvet M, Endo I, Hoffman RM (2016). Patient-derived mouse models of cancer need to be orthotopic in order to evaluate targeted anti-metastatic therapy. Oncotarget.

[41] Murakami T, Murata T, Kawaguchi K, Kiyuna T, Igarashi K, Hwang HK, Hiroshima Y, Hozumi C, Komatsu S, Kikuchi T, Lwin TM, Delong JC, Miyake K (2017). Cervical cancer patient-derived orthotopic xenograft (PDOX) is sensitive to cisplatinum and resistant to nab-paclitaxel. Anticancer Re.

[42] Yamamoto M, Zhao M, Hiroshima Y, Zhang Y, Shurell E, Eilber FC, Bouvet M, Noda M, Hoffman RM (2016). Efficacy of tumor-targeting Salmonella typhimurium A1-R on a melanoma patient-derived orthotopic xenograft (PDOX) nude-mouse model. Plos One.

[43] Kawaguchi K, Murakami T, Chmielowski B, Igarashi K, Kiyuna T, Unno M, Nelson SD, Russell TA, Dry SM, Li Y, Eilber FC, Hoffman RM (2016). Vemurafenib-resistant BRAF-V600E mutated melanoma is regressed by MEK targeting drug trametinib, but not cobimetinib in a patient-derived orthotopic xenograft (PDOX) mouse model. Oncotarget.

[44] Kawaguchi K, Igarashi K, Murakami T, Chmielowski B, Kiyuna T, Zhao M, Zhang Y, Singh A, Unno M, Nelson SD, Russell TA, Dry SM, Li Y (2016). Tumor-targeting Salmonella typhimurium A1-R combined with temozolomide regresses malignant melanoma with a BRAF-V600E mutation in a patient-derived orthotopic xenograft (PDOX) model. Oncotarget.

[45] Guo HY, Herrera H, Groce A, Hoffman RM (1993). Expression of the biochemical defect of methionine dependence in fresh patient tumors in primary histoculture. Cancer Res.

[46] Yano S, Takehara K, Zhao M, Tan Y, Han Q, Li S, Bouvet M, Fujiwara T, Hoffman RM (2016). Tumor-specific cell-cycle decoy by Salmonella typhimurium A1-R combined with tumor-selective cell-cycle trap by methioninase overcome tumor intrinsic chemoresistance as visualized by FUCCI imaging. Cell Cycle.

[47] Yano S, Li S, Han Q, Tan Y, Bouvet M, Fujiwara T, Hoffman RM (2014). Selective methioninase-induced trap of cancer cells in S/G2 phase visualized by FUCCI imaging confers chemosensitivity. Oncotarget.

[48] Yano S, Zhang Y, Zhao M, Hiroshima Y, Miwa S, Uehara F, Kishimoto H, Tazawa H, Bouvet M, Fujiwara T, Hoffman RM (2014). Tumor-targeting Salmonella typhimurium A1-R decoys quiescent cancer cells to cycle as visualized by FUCCI imaging and become sensitive to chemotherapy. Cell Cycle.

[49] Tan Y, Xu M, Tan XZ, Tan XY, Wang X, Saikawa Y, Nagahama T, Sun X, Lenz M, Hoffman RM (1997). Overexpression and large-scale production of recombinant L-methionine-α-deamino-γ-mercaptomethane-lyase for novel anticancer therapy. Prot Exp Purification.

[50] Sun X, Tan Y, Yang Z, Li S, Hoffman RM (2005). A rapid HPLC method for the measurement of ultra-low plasma methionine concentrations applicable to methionine depletion therapy. Anticancer Res.

[51] Blagosklonny MV (2003). Matching targets for selective cancer therapy. Drug Discov Today.

[52] Blagosklonny MV (2005). Teratogens as anti-cancer drugs. Cell Cycle.

[53] Blagosklonny MV (2001). Treatment with inhibitors of caspases, that are substrates of drug transporters, selectively permits chemotherapy-induced apoptosis in multidrug-resistant cells but protects normal cells. Leukemia.

[54] Blagosklonny MV (2006). Target for cancer therapy: proliferating cells or stem cells. Leukemia.

[55] Apontes P, Leontieva OV, Demidenko ZN, Li F, Blagosklonny MV (2011). Exploring long-term protection of normal human fibroblasts and epithelial cells from chemotherapy in cell culture. Oncotarget.

[56] Blagosklonny MV (2003). Tissue-selective therapy of cancer. Br J Cancer.

[57] Hoffman RM, Jacobsen SJ, Erbe RW (1978). Reversion to methionine independence by malignant rat and SV40-transformed human fibroblasts. Biochem Biophys Res Commun.

[58] Hoffman RM, Jacobsen SJ (1980). Reversible growth arrest in simian virus 40-transformed human fibroblasts. Proc Natl Acad Sci USA.

